# Sex differences in susceptibility, severity, and outcomes of coronavirus disease 2019: Cross-sectional analysis from a diverse US metropolitan area

**DOI:** 10.1371/journal.pone.0245556

**Published:** 2021-01-13

**Authors:** Farhaan S. Vahidy, Alan P. Pan, Hilda Ahnstedt, Yashasvee Munshi, Huimahn A. Choi, Yordanos Tiruneh, Khurram Nasir, Bita A. Kash, Julia D. Andrieni, Louise D. McCullough

**Affiliations:** 1 Center for Outcomes Research, Houston Methodist Research Institute, Houston, TX, United States of America; 2 Houston Methodist Neurological Institute, Houston Methodist, Houston, TX, United States of America; 3 Department of Neurology, McGovern Medical School, UTHealth, Houston, TX, United States of America; 4 Department of Neurosurgery, McGovern Medical School, UTHealth, Houston, TX, United States of America; 5 University of Texas Health Science Center, Tyler, TX, United States of America; 6 Department of Cardiology, Houston Methodist, Houston, TX, United States of America; 7 Texas A&M, School of Rural Public Health, College Station, TX, United States of America; 8 Weill Cornell Medical College, New York, NY, United States of America; 9 Department of Clinical Medicine, Houston Methodist, Houston, TX, United States of America; Azienda Ospedaliero Universitaria Careggi, ITALY

## Abstract

**Introduction:**

Sex is increasingly recognized as an important factor in the epidemiology and outcome of many diseases. This also appears to hold for coronavirus disease 2019 (COVID-19). Evidence from China and Europe has suggested that mortality from COVID-19 infection is higher in men than women, but evidence from US populations is lacking. Utilizing data from a large healthcare provider, we determined if males, as compared to females have a higher likelihood of SARS-CoV-2 susceptibility, and if among the hospitalized COVID-19 patients, male sex is independently associated with COVID-19 severity and poor in-hospital outcomes.

**Methods and findings:**

Using the Strengthening the Reporting of Observational Studies in Epidemiology (STROBE) guidelines, we conducted a cross-sectional analysis of data from a COVID-19 Surveillance and Outcomes Registry (CURATOR). Data were extracted from Electronic Medical Records (EMR). A total of 96,473 individuals tested for SARS-CoV-2 RNA in nasopharyngeal swab specimens via Polymerized Chain Reaction (PCR) tests were included. For hospital-based analyses, all patients admitted during the same time-period were included. Of the 96,473 patients tested, 14,992 (15.6%) tested positive, of whom 4,785 (31.9%) were hospitalized and 452 (9.5%) died. Among all patients tested, men were significantly older. The overall SARS-CoV-2 positivity among all tested individuals was 15.5%, and was higher in males as compared to females 17.0% vs. 14.6% [OR 1.20]. This sex difference held after adjusting for age, race, ethnicity, marital status, insurance type, median income, BMI, smoking and 17 comorbidities included in Charlson Comorbidity Index (CCI) [aOR 1.39]. A higher proportion of males (vs. females) experienced pulmonary (ARDS, hypoxic respiratory failure) and extra-pulmonary (acute renal injury) complications during their hospital course. After adjustment, length of stay (LOS), need for mechanical ventilation, and in-hospital mortality were significantly higher in males as compared to females.

**Conclusions:**

In this analysis of a large US cohort, males were more likely to test positive for COVID-19. In hospitalized patients, males were more likely to have complications, require ICU admission and mechanical ventilation, and had higher mortality than females, independent of age. Sex disparities in COVID-19 vulnerability are present, and emphasize the importance of examining sex-disaggregated data to improve our understanding of the biological processes involved to potentially tailor treatment and risk stratify patients.

## Introduction

As the Coronavirus Disease (COVID-19) pandemic continues to unfold and evolve across the globe, population sub-groups with higher levels of disease vulnerability have been identified. The risk stratification for either contracting the severe acute respiratory syndrome coronavirus 2 (SARS-CoV-2) or outcomes associated with COVID-19 are important for developing targeted prevention and management strategies. Advanced age and pre-existing cardiac and metabolic conditions have now been widely reported to be associated with poor outcomes [1, 2]. Furthermore, higher susceptibility related to minority race and ethnicity driven by social determinants have also come to light [3]. The evidence for potential sex differences in the COVID-19 pandemic is continuing to emerge. Initial small studies from China indicated that males tend to experience higher disease severity as compared to females; and that among males, individuals with comorbidities had a higher likelihood of critical illness, however a similar association was not observed for females [4, 5]. This study was followed by several narrative reviews that summarized publicly available epidemiological data from Europe and China and speculated various behavioral (gender factors) and biological pathways (sex factors) that may be responsible for sex differences related to COVID-19 [6, 7]. More recently, further preliminary evidence of differential immune response to SARS-CoV-2 between males and females has been elucidated [8]. While gender-related behaviors such as smoking, drinking, the propensity to seek hospital care and presence of comorbidities could affect the outcome of COVID-19, the increased risk of death seen in males across several different cultures in the world point to biological risk determinants. Despite these preliminary studies, a comprehensive analysis of sex differences in the epidemiological features of COVID-19 disease from a large and diverse United States (US) metropolitan area is lacking. More specifically, it has not been demonstrated if males have an independently higher risk of contracting SARS-CoV-2 and if males, in comparison to females, experience higher COVID-19 severity leading to poor outcomes.

Houston is the fourth largest and the most diverse metropolitan area in the US. Following an initial surge in COVID-19 cases during March and April 2020, the greater Houston metropolitan area experienced a larger magnitude of COVID-19 resurgence between June and August 2020 [9]. Utilizing data from a large healthcare provider in the Houston metropolitan area, we aimed to determine if males, as compared to females have a higher likelihood of SARS-CoV-2 susceptibility, and if among the hospitalized COVID-19 patients, male sex is interpedently associated with COVID-19 severity and poor in-hospital outcomes.

## Methods

### Study setting and design

Using the Strengthening the Reporting of Observational Studies in Epidemiology (STROBE) guidelines, we conducted a cross-sectional analysis of data from the Houston Methodist COVID-19 Surveillance and Outcomes Registry (CURATOR). The Houston Methodist is an 8-hospital tertiary healthcare system serving the diverse greater Houston population. The Houston Methodist CURATOR is an Institutional Review Board (IRB) approved observational research registry for COVID-19 patients that tracks socio-demographic, comorbidity, clinical and outcomes data on all individuals tested for SARS-CoV-2 across Houston Methodist. Data are extracted from Electronic Medical Records (EMR) and are continually assessed for quality, consistency and validity. Adult (≥ 18 years), male and female individuals of all races and ethnicities who were tested between March 6 and August 22, 2020, for the presence of SARS-CoV-2 RNA in nasopharyngeal swab specimens via Polymerized Chain Reaction (PCR) tests were included. For hospital-based analyses, all patients admitted to any of the eight Houston Methodist hospital across greater Houston during the same time-period were included. Sex, either male or female, as self-identified by patients at the time of testing or hospital admission was the exposure of interest.

### Outcomes

For susceptibility analyses positive SARS-CoV-2 PCR test was regarded as the outcome. Patients who underwent repeat testing were considered positive if at least one test was positive and were considered negative if all tests were negative. Analyses were conducted at the patient level and information from the first (positive or negative) encounter was utilized. For hospital-based analyses, we analyzed admission to the Intensive Care Unit (ICU), need for mechanical ventilation, and hospital length of stay (LOS) as markers of disease severity. Throughout the reporting time period, hospital and ICU admission guidelines were consistently based on risk stratification by evaluation of severity of symptoms, comorbidities, diagnostic findings, pulse oximetry and need for specialized procedures such as mechanical ventilation. In-hospital mortality was analyzed as a poor COVID-19 outcome among hospitalized patients.

### Other covariates

We included socio-demographic (age, race, ethnicity, marital status, insurance, Zip code based median income and population density), behavioral (smoking), and comorbidity (body mass index, hypertension, hyperlipidemia, asthma, myocardial infarction, congestive heart failure, peripheral vascular disease, cerebrovascular disease, dementia, chronic pulmonary disease, liver disease, renal disease, diabetes with our without complications, paraplegia/hemiplegia, cancer, and HIV/AIDS) variables in our analyses. All variables were derived from EMR into CURATOR as per pre-specified data definitions. Seventeen Comorbidities comprising the Charlson Comorbidity Index (CCI) were used to determine patient-level CCI for certain analyses [10]. We used the US Census Bureau’s American Community Survey 5-year data (2014–2018) to determine the median household income by individual zip code tabulation area (ZCTA) [11]. The median ZCTA household income was inflation adjusted to 2018 US dollars. We also used the same data source to obtain population estimates by ZCTA, and calculated ZCTA-level population density (population per mile square) by standardizing if for area measurements of ZCTA. For the purpose of population density determination, land area estimates were obtained from the Census Bureau’s US Gazetteer Files 2010 [12]. For hospital analyses, we included vital signs (Systolic and Diastolic Blood Pressure, Respiratory Rate, Temperature, Oxygen Saturation), laboratory values (White Cell Count, Lymphocytes, Platelet Count, Liver Enzymes, Bilirubin, Creatinine, D-Dimer, Ferritin, Venous Lactate, C-Reactive Protein, and Troponin) and pulmonary (pneumonia, acute respiratory distress syndrome [ARDS], bronchitis, hypoxic respiratory failure) and extra-pulmonary (acute renal / hepatic injury, congestive heart failure) complications. We also compared utilization of medications such as hydroxychloroquine, Remdesivir, Dexamethasone, Antithrombotics, and Anticoagulants between male and female COVID-19 patients.

### Statistical analyses

A descriptive account of male and female individuals who were tested for SARS-CoV-2 RNA via PCR, and who were hospitalized for COVID-19 is provided as proportions and 95% confidence intervals (CI) around the proportions. Univariable analyses were conducted using t-test or chi-square tests as appropriate for the functional form of data elements. We fit multivariable regression models to assess for independent association between sex and pre-specific outcomes. Separate logistic regression models were fitted to determine the likelihood of SARS-CoV-2 infectivity, admission to ICU, utilization of mechanical ventilation and in-hospital mortality among male and female individuals. Model fit and discrimination was assessed by Hosmer Lemeshow goodness of fit test and by assessment of area under the receiver operating curve (AUC). Unadjusted and adjusted Odds Ratios (aOR) along with 95% confidence intervals are provided. We fit quantile regression models to provide adjusted estimates of difference in median length of hospital stay between male and female COVID-19 patients. Coefficients (CI) of this model represent the median difference in LOS. Variables to be included in regression models were selected based on statistical (p < 0.1) and pre-determined clinical significance. Functional form of co-variates was transformed for certain analyses to achieve optimal model fit. Variables with > 10% missing information were excluded from multivariable models. All analyses were conducted with STATA (v.16 STAT Corp Austin, TX) and the level of significance was set at 0.05.

## Results

Between March 2 and August 22, 2020, a total of 96,496 individuals were tested for the presence of SARS-CoV-2 RNA. Information on sex could not be determined for 23 (0.02%) individuals. From among the 96,473 individuals included 14,992 (15.6%) tested positive, of whom 4,785 (31.9%) were hospitalized and 452 (9.5%) died.

### Sex differences among the tested and hospitalized cohorts

In the tested cohort (n = 96,473) males (vs. females) were significantly older (mean age 53.3 vs. 49.8 years) and predominantly white (66.7% vs. 64.0%). Consequently, among females (vs. males) there was a greater proportion of individuals identifying with Black race (21.0% vs. 16.5%) and Hispanic ethnicity (21.3% vs. 19.8%). As compared to males, a greater proportion of females were single (31.0% vs. 27.7%) and were commercially insured (51.2% vs. 43.8%). The overall comorbidity burden was higher among males, CCI median IQR for males (vs. females) was 2 (0–5) vs. 1 (0–4) (p < 0.001). Though the proportion of obesity was higher among females (29.4% vs. 28.4%), a higher proportion of males were current or past smokers (41.8% vs. 24.8%). The comorbidity burden for males was driven by greater proportions for cardio-vascular disease (hypertension, myocardial infarction, congestive heart failure, peripheral vascular disease), cerebrovascular disease, diabetes, cancer and HIV/AIDS. Univariable comparison between males and females across various socio-demographic and comorbidity variables is provided in Table 1.

**Table 1:** Socio-demographic and comorbidity differences between male and female individuals tested for SARS-CoV-2 at Houston Methodist.

	Total (96,473)	Female (57,483)	Male (38,990)	p-value
**Demographic and Social Characteristics, n (%)**				
Age, mean (SD)	51.2 (18.6)	49.8 (18.5)	53.3 (18.5)	<0.001
Race				<0.001
White	62,806 (65.1)	36,790 (64.0)	26,016 (66.7)	
Black	18,540 (19.2)	12,098 (21.0)	6,442 (16.5)	
Asian	5,802 (6.0)	3,611 (6.3)	2,191 (5.6)	
Other	9,325 (9.7)	4,984 (8.7)	4,341 (11.1)	
Hispanic	19,022 (20.7)	11,801 (21.3)	7,221 (19.8)	<0.001
Marital Status				<0.001
Single	28,590 (29.6)	17,805 (31.0)	10,785 (27.7)	
Partner	52,245 (54.2)	29,295 (51.0)	22,950 (58.9)	
Separated	10,939 (11.3)	8,166 (14.2)	2,773 (7.1)	
Unavailable	4,699 (4.9)	2,217 (3.9)	2,482 (6.4)	
Insurance				<0.001
Commercial	46,536 (48.2)	29,458 (51.2)	17,078 (43.8)	
Medicare	26,977 (28.0)	14,735 (25.6)	12,242 (31.4)	
Medicaid	3,835 (4.0)	3,087 (5.4)	748 (1.9)	
Self-Pay	16,839 (17.5)	8,992 (15.6)	7,847 (20.1)	
Other	2,286 (2.4)	1,211 (2.1)	1,075 (2.8)	
Median Income, median (IQR)	70,324 (53313–99276)	70,444 (53313–99276)	70,324 (53263–99276)	0.80
Residence Below Poverty Level	41,269 (43.2)	24,534 (43.1)	16,735 (43.4)	0.41
Residence in High Population Density Zip	37,698 (39.5)	22,208 (39.0)	15,490 (40.2)	<0.001
**Comorbidities / Risk Factors / Pre-existing Conditions, n (%)**				
Charlson Comorbidity Index, median (IQR)	2 (0–5)	1 (0–4)	2 (0–5)	<0.001
Obesity	27,923 (29.0)	16,868 (29.4)	11,055 (28.4)	<0.001
Smoking (Current/Past)	25,257 (31.4)	12,148 (24.8)	13,109 (41.8)	<0.001
Hypertension	44,171 (45.8)	24,054 (41.9)	20,117 (51.6)	<0.001
Hyperlipidemia	34,213 (35.5)	18,128 (31.5)	16,085 (41.3)	<0.001
Asthma	11,240 (11.7)	8,042 (14.0)	3,198 (8.2)	<0.001
Myocardial Infarction	7,878 (8.2)	3,470 (6.0)	4,408 (11.3)	<0.001
Congestive Heart Failure	10,738 (11.1)	5,276 (9.2)	5,462 (14.0)	<0.001
Peripheral Vascular Disease	10,853 (11.3)	5,227 (9.1)	5,626 (14.4)	<0.001
Cerebrovascular Disease	10,252 (10.6)	5,308 (9.2)	4,944 (12.7)	<0.001
Dementia	3,072 (3.2)	1,712 (3.0)	1,360 (3.5)	<0.001
Chronic Pulmonary Disease	20,027 (20.8)	12,908 (22.5)	7,119 (18.3)	<0.001
Rheumatic Disease	3,509 (3.6)	2,846 (5.0)	663 (1.7)	<0.001
Peptic Ulcer Disease	3,175 (3.3)	1,931 (3.4)	1,244 (3.2)	0.15
Liver Disease (Mild)	8,208 (8.5)	4,680 (8.1)	3,528 (9.1)	<0.001
Liver Disease (Moderate to Severe)	1,301 (1.3)	604 (1.1)	697 (1.8)	<0.001
Diabetes Without Complications	19,500 (20.2)	10,312 (17.9)	9,188 (23.6)	<0.001
Diabetes With Complications	7,283 (7.6)	3,451 (6.0)	3,832 (9.8)	<0.001
Hemiparesis	1,721 (1.8)	883 (1.5)	838 (2.2)	<0.001
Renal Disease	4,244 (4.4)	1,992 (3.5)	2,252 (5.8)	<0.001
Cancer	9,563 (9.9)	5,089 (8.9)	4,474 (11.5)	<0.001
Carcinoma (Metastatic)	7,321 (7.6)	4,104 (7.1)	3,217 (8.3)	<0.001
AIDS or HIV	608 (0.6)	201 (0.3)	407 (1.0)	<0.001

Missing variables: Hispanic (6.7%), Median Income (1.2%), Poverty (1.0%), Population Density (1.0%), Smoking (16.7%), CCI comorbidities (> 0.1%).

In the hospitalization cohort, there were no significant differences between males and females for age (59.1 vs. 59.3 years) or ethnicity (Hispanic: 39.3% vs. 37.4%). However, as compared to males, a greater proportion of females in the hospitalization cohort identified with Black race (29.6% vs. 22.2%) and had lower median Zip-based income (US $ 60,130 vs. US $ 64,648). Similar to the testing cohort, among hospitalized COVID-19 patients, females (vs. males) had a higher mean BMI (32.2 kg/m^2^ vs. 30.5 kg/m^2^) and a greater proportion of males (vs. females) were current or past smokers (32.9% vs. 20.2%). The burden of cardiovascular disease was higher among males hospitalized for COVID-19 (myocardial infarction 19.5% vs. 15.7% for females, congestive heart failure 21.7% vs. 19.9% for females, and peripheral vascular disease 16.1% vs. 13.4% for females). A higher proportion of females (vs. males) had pre-existing rheumatic disease (5.6% vs. 2.0%), chronic pulmonary disease (26.3% vs. 19.2%), mild cognitive impairment / dementia (11.5% vs. 8.9%) and other neurological conditions such as epilepsy, Parkinson’s disease and Motor Neuron Disease (9.9% vs. 7.7%). There were no statistically significant sex differences observed for pre-existing cerebrovascular disease and stroke. Likewise, the overall CCI was not different for males and females, CCI median (IQR): 3 (1–7). A detailed comparison between males and females hospitalized for COVID-19 across various socio-demographic and comorbidity variables is provided in Table 2. A greater portion of males experienced tachypnea, pyrexia and hypoxemia during hospitalization. Overall, the proportion of derangement in laboratory parameters were higher among males (vs. females) hospitalized for COVID-19. This was predominantly manifested by lymphocytopenia, thrombocytopenia and elevated levels of pro-calcitonin, aspartate and alanine transaminases, total bilirubin, creatinine, C-Reactive protein, serum ferritin and venous lactate. A higher proportion of males (vs. females) also experienced pulmonary (ARDS, hypoxic respiratory failure) and extra-pulmonary (acute renal injury) complications during their hospital course. Detailed comparison between COVID-19 hospitalized males and females across various hospitalization variables such as vital signs, laboratory parameters, in-hospital complications and medications are provided in Table 2.

**Table 2:** Socio-demographic and comorbidity differences between male and female COVID-19 patients admitted at Houston Methodist.

	Total (4,785)	Female (2,339)	Male (2,446)	p-value
**Demographic and Social Characteristics, n (%)**				
Age (Years)	59.2 (17.3)	59.3 (18.4)	59.1 (16.2)	0.65
Race				<0.001
White	2,950 (61.7)	1,377 (58.9)	1,573 (64.3)	
Black	1,234 (25.8)	692 (29.6)	542 (22.2)	
Asian	224 (4.7)	92 (3.9)	132 (5.4)	
Other	377 (7.9)	178 (7.6)	199 (8.1)	
Ethnicity	1,811 (38.4)	864 (37.4)	947 (39.3)	0.18
Marital Status				<0.001
Single	1,352 (28.3)	719 (30.7)	633 (25.9)	
Married / Partner	2,534 (53.0)	1,019 (43.6)	1,515 (61.9)	
Widowed / Separated	745 (15.6)	529 (22.6)	216 (8.8)	
Unknown	154 (3.2)	72 (3.1)	82 (3.4)	
Insurance Type				<0.001
Commercial	1,604 (33.5)	730 (31.2)	874 (35.7)	
Medicaid	282 (5.9)	190 (8.1)	92 (3.8)	
Medicare	1,833 (38.3)	938 (40.1)	895 (36.6)	
Other	260 (5.4)	132 (5.6)	128 (5.2)	
Self-Pay	806 (16.8)	349 (14.9)	457 (18.7)	
Median Zip-based Income	62,083 (47,303–79,658)	60,130 (46,801–78,052)	64,648 (47,817–79,869)	<0.001
Residence Below Poverty Level (Census Track)	2,758 (58.1)	1,383 (59.7)	1,375 (56.5)	0.027
Population Density (High vs. Low)	2,089 (44.0)	1,026 (44.3)	1,063 (43.7)	0.69
**Comorbidities / Risk Factors / Pre-existing conditions, n (%)**				
Body Mass Index, mean (SD)	31.3 (8.2)	32.2 (8.7)	30.5 (7.7)	<0.001
Obesity	1,736 (36.3)	900 (38.5)	836 (34.2)	0.002
Smoking (Current / Past)	1,188 (26.7)	444 (20.2)	744 (32.9)	<0.001
Hypertension	3,268 (68.3)	1,569 (67.1)	1,699 (69.5)	0.077
Hyperlipidemia	2,189 (45.7)	1,030 (44.0)	1,159 (47.4)	0.020
Asthma	508 (10.6)	349 (14.9)	159 (6.5)	<0.001
Myocardial Infarction	844 (17.6)	367 (15.7)	477 (19.5)	<0.001
Congestive Heart Failure	995 (20.8)	465 (19.9)	530 (21.7)	0.13
Peripheral Vascular Disease	707 (14.8)	314 (13.4)	393 (16.1)	0.010
Cerebrovascular Disease	748 (15.6)	367 (15.7)	381 (15.6)	0.91
Dementia	472 (9.9)	264 (11.3)	208 (8.5)	0.001
Chronic Pulmonary Disease	1,085 (22.7)	616 (26.3)	469 (19.2)	<0.001
Rheumatic Disease	181 (3.8)	132 (5.6)	49 (2.0)	<0.001
Peptic Ulcer Disease	146 (3.1)	77 (3.3)	69 (2.8)	0.34
Mild Liver Disease	442 (9.2)	204 (8.7)	238 (9.7)	0.23
Moderate to Severe Liver Disease	92 (1.9)	41 (1.8)	51 (2.1)	0.40
Diabetes Without Complications	2,038 (42.6)	981 (41.9)	1,057 (43.2)	0.37
Diabetes With Complications	867 (18.1)	400 (17.1)	467 (19.1)	0.074
Paraplegia / Hemiplegia	128 (2.7)	56 (2.4)	72 (2.9)	0.24
Renal Disease	482 (10.1)	231 (9.9)	251 (10.3)	0.66
Cancer	421 (8.8)	185 (7.9)	236 (9.6)	0.034
Metastatic Carcinoma	314 (6.6)	163 (7.0)	151 (6.2)	0.27
AIDS / HIV	41 (0.9)	13 (0.6)	28 (1.1)	0.027
Stroke (Ischemic / Hemorrhagic)	515 (10.8)	256 (10.9)	259 (10.6)	0.69
Mild Cognitive Impairment / Dementia	486 (10.2)	269 (11.5)	217 (8.9)	0.003
Other Neurological Disorders	419 (8.8)	231 (9.9)	188 (7.7)	0.007
Charlson Comorbidity Index, median (IQR)	3 (1–7)	3 (1–7)	3 (1–7)	0.76
**Vital Signs**				
Systolic Blood Pressure	132.7 (19.9)	132.3 (21.0)	133.1 (18.8)	0.19
Diastolic Blood Pressure	72.4 (10.0)	70.4 (9.6)	74.2 (10.0)	<0.001
Respiratory Rate ≥ 24 breaths / min	961 (21.5)	409 (18.7)	552 (24.1)	<0.001
Temperature ≥ 38 C	212 (4.7)	89 (4.1)	123 (5.4)	0.041
**Laboratory Parameters**				
Oxygen Saturation < 94%	806 (18.1)	305 (14.0)	501 (21.9)	<0.001
White Blood Cell Count < 4000/microliter	264 (5.5)	154 (6.6)	110 (4.5)	0.001
Lymphocytes < 20%	3,254 (68.2)	1,442 (61.9)	1,812 (74.3)	<0.001
Platelet Count 150,000 / microliter	549 (11.5)	222 (9.5)	327 (13.4)	<0.001
B-natriuretic peptide > 100 pg/ml	1,176 (34.3)	576 (35.1)	600 (33.5)	0.34
Procalcitonin > 0.25 ng/ml	848 (47.6)	331 (43.7)	517 (50.4)	0.005
Troponin ≥ 0.06 ng/ml	922 (33.2)	403 (32.5)	519 (33.7)	0.50
Aspartate aminotransferase > 40 U/l	2,304 (49.3)	933 (41.4)	1,371 (56.6)	<0.001
Alanine aminotransferase > 40 U/l	1,970 (42.3)	704 (31.3)	1,266 (52.4)	<0.001
Total Bilirubin ≥ 1.2 mg / dl	181 (4.1)	62 (3.1)	119 (5.1)	<0.001
C-Reactive Protein >8.2 ng/ml	3,855 (94.4)	1,788 (92.7)	2,067 (95.9)	<0.001
Ferritin level > 3000 ng/ml	233 (5.6)	82 (4.2)	151 (7.0)	<0.001
D-dimer > 0.5 ug/ml	3,444 (84.8)	1,629 (84.1)	1,815 (85.4)	0.26
Creatinine > 1.5 mg/dl	821 (17.4)	330 (14.5)	491 (20.1)	<0.001
Venous lactate > 2.2 mmol/l	740 (21.6)	312 (19.3)	428 (23.6)	0.002
**Hospital Conditions / Complications**				
Pneumonia	3,884 (81.2)	1,844 (78.8)	2,040 (83.4)	<0.001
Acute Respiratory Distress Syndrome	444 (9.3)	184 (7.9)	260 (10.6)	<0.001
Bronchitis	127 (2.7)	73 (3.1)	54 (2.2)	0.049
Lower Respiratory Tract Infection	99 (2.1)	61 (2.6)	38 (1.6)	0.010
Acute Renal Injury	1,632 (34.1)	688 (29.4)	944 (38.6)	<0.001
Acute Hepatic Injury	129 (2.7)	64 (2.7)	65 (2.7)	0.87
Congestive Heart Failure	895 (18.7)	423 (18.1)	472 (19.3)	0.28
Hypoxic Respiratory Failure	2,595 (54.2)	1,202 (51.4)	1,393 (57.0)	<0.001
**Medications, n (%)**				
Hydroxychloroquine	374 (7.8)	180 (7.7)	194 (7.9)	0.76
Ribavirin	104 (2.2)	46 (2.0)	58 (2.4)	0.34
Azithromycin	721 (15.1)	348 (14.9)	373 (15.2)	0.72
Lopinavir / Ritonavir	25 (0.5)	9 (0.4)	16 (0.7)	0.20
Remdesivir	1,048 (21.9)	424 (18.1)	624 (25.5)	<0.001
Tocilizumab	800 (16.7)	290 (12.4)	510 (20.9)	<0.001
Antithrombotic	1,651 (34.5)	752 (32.2)	899 (36.8)	<0.001
Anticoagulants	4,331 (90.5)	2,072 (88.6)	2,259 (92.4)	<0.001
Dexamethasone	2,370 (49.5)	1,073 (45.9)	1,297 (53.0)	<0.001
Donepezil	145 (3.0)	81 (3.5)	64 (2.6)	0.088
Rivastigmine	31 (0.6)	15 (0.6)	16 (0.7)	0.96
Galantamine	8 (0.2)	5 (0.2)	3 (0.1)	0.44
Memantine	111 (2.3)	64 (2.7)	47 (1.9)	0.061
**Outcomes**				
ICU Admit	1,480 (30.9)	646 (27.6)	834 (34.1)	<0.001
In-patient Length of Stay (Days)	5 (2–9)	4 (2–8)	5 (2–9)	<0.001
Mechanical Ventilation	809 (16.9)	344 (14.7)	465 (19.0)	<0.001
In-Hospital Mortality	452 (9.4)	185 (7.9)	267 (10.9)	<0.001

### Association between sex and outcomes | SARS-CoV-2 positivity, intensive care unit admission, mechanical ventilation, in-hospital mortality and length of stay

**Fig 1:**
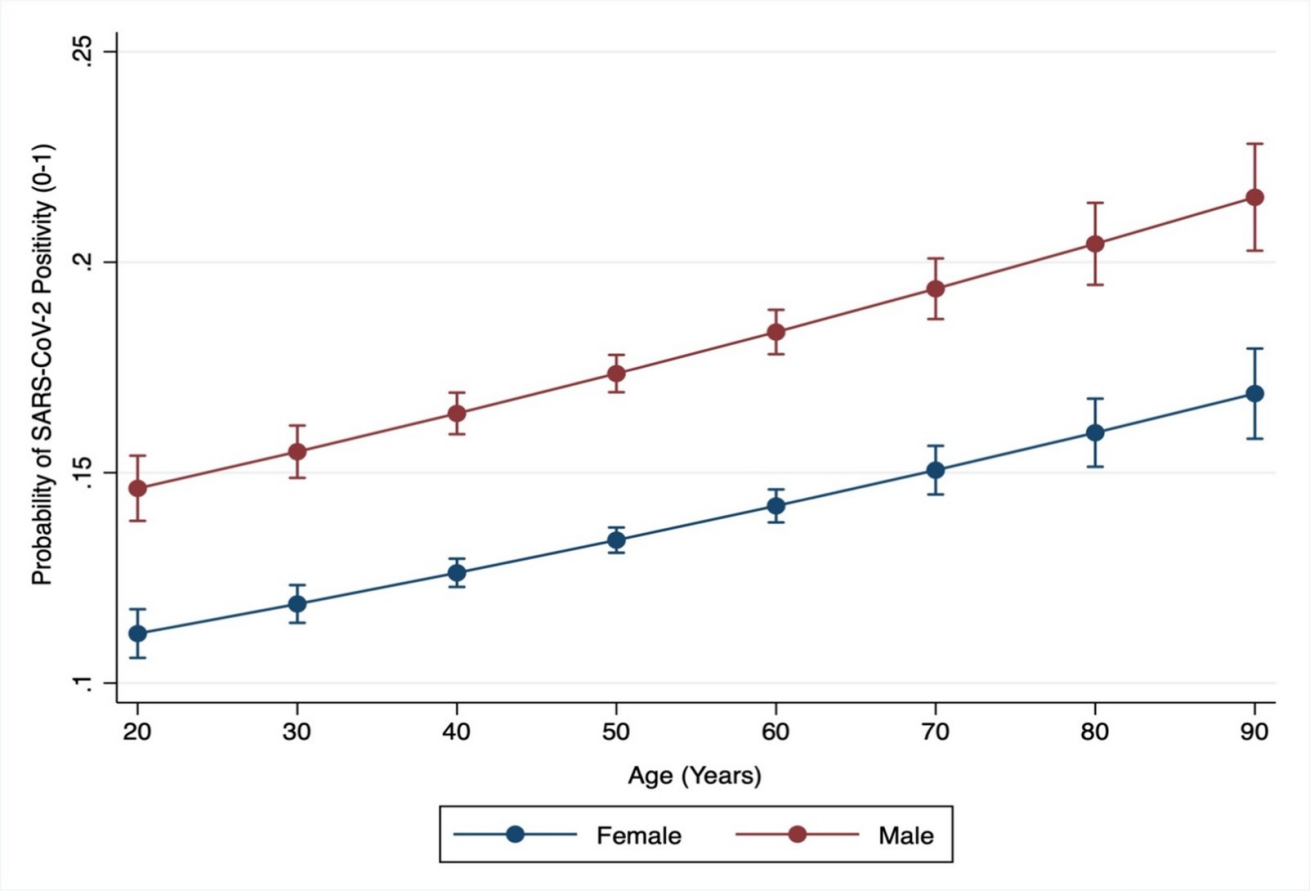
Predicted probabilities of SARS-CoV-2 positivity (Y-axis) by age (X-axis) among males and females. Predicted probably of SARS-CoV-2 infection for males (red line) and females (blue line) for age spectrum (20–90 years). Probability demonstrated on a scale of 0–1.

The overall proportion (CI) for SARS-CoV-2 positivity among all tested individuals was 15.5% (15.3–15.8). The proportion (CI) among males was significantly higher as compared to females 17.0% (16.6–17.3) vs. 14.6% (14.3–14.9) [OR(CI): 1.20 (1.16–1.24)]. After adjusting for age, race, ethnicity, marital status, insurance type, median income, BMI, smoking and 17 comorbidities included in CCI, males had a significantly higher likelihood of SARS-CoV-2 positivity as compared to females aOR (CI):1.39 (1.33–1.45). There was no interaction between sex and age for SARS-CoV-2 positivity, and a statistically significant higher likelihood of SARS-CoV-2 positivity among males was observed across all ages. Based on the fully adjusted model, the likelihood of SARS-CoV-2 positivity for males and females across the age spectrum is demonstrated in Fig 1.

**Fig 2:**
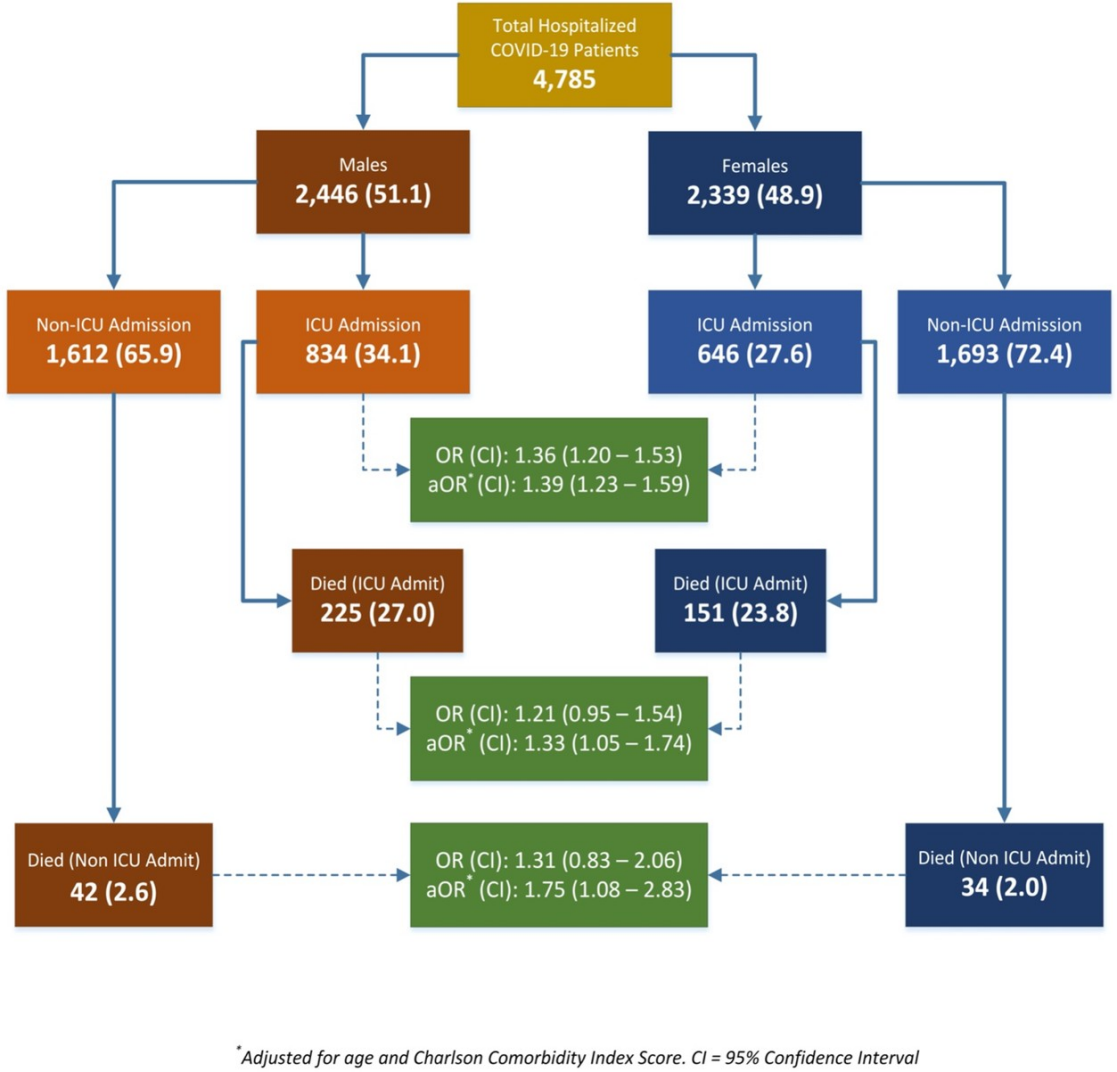
Frequency and proportion of hospital outcomes among COVID-19 patients, by sex. OR: Odds Ratio, aOR: Adjusted Odds Ratio, CI: 95% Confidence Interval, Respective ORs and 95% (CI) represent odds of ICU admission and death for males (vs. females) among hospitalized COVID-19 patients.

In the COVID-19 hospitalization cohort, the overall proportion (CI) of patients who were managed in the ICU was 30.9% (29.6–32.3). This proportion was significantly higher among males 34.1% (32.2–36.0) as compared to females 27.6% (25.8–29.5), OR (CI): 1.36 (1.20–1.53). The significantly higher likelihood of ICU admission among males persisted after adjusting for pre-hospitalization socio-demographic and comorbidity variables, aOR (CI): 1.31 (1.13–1.51). Among the hospitalized COVID-19 patients, 16.9% (15.9–18.0) required invasive mechanical ventilation (54.7% among patients admitted to the ICU). The proportion of males who underwent mechanical ventilation was 19.0% (17.5–20.6), which was significantly higher than females 14.7% (13.3–16.2), OR (CI): 1.36 (1.17–1.59). Similar to the ICU admission estimate, the adjusted estimate for mechanical ventilation continued to be significantly higher for males vs. females, aOR (CI): 1.29 (1.08–1.55). At the time of our analyses, 276 (5.8%) patients were still hospitalized. We excluded these patients from analyses related to in-hospital mortality. From among the patients who had either been discharged or died (n = 4,509) a total of 452 [10.0% (9.2–10.9)] experienced in-hospital mortality. The proportion (CI) of males who experienced in-hospital mortality 11.6% (10.4–13.0) was significantly higher as compared to females 8.3% (7.3–9.6), OR (CI): 1.44 (1.18–1.75). Guided by our analysis of pre-hospital and hospital factors associated with mortality (S1 Table) we fitted a fully adjusted logistic regression model to assess the independent association between sex and in-hospital mortality. Based on our final model adjusted for age, insurance, comorbidities (myocardial infarctions, peptic ulcer disease, diabetes), total Charlson Comorbidity Index, smoking, respiratory rate, oxygen saturation, White Blood Count, Platelet Count, Creatinine levels, pulmonary (ARDS) and extra-pulmonary (acute renal and hepatic injury) complications, ICU stay and requirement of mechanical ventilation, the likelihood of in-hospital mortality was independently and significantly higher among males as compared to females, aOR (CI): 1.45 (1.06–2.00). The overall and sex specific proportions, crude and adjusted (for age and CCI) ORs and CI for SARS-CoV-2 infection, ICU admission and mortality among ICU and non-ICU patients are schematically represented in Fig 2.

**Fig 3:**
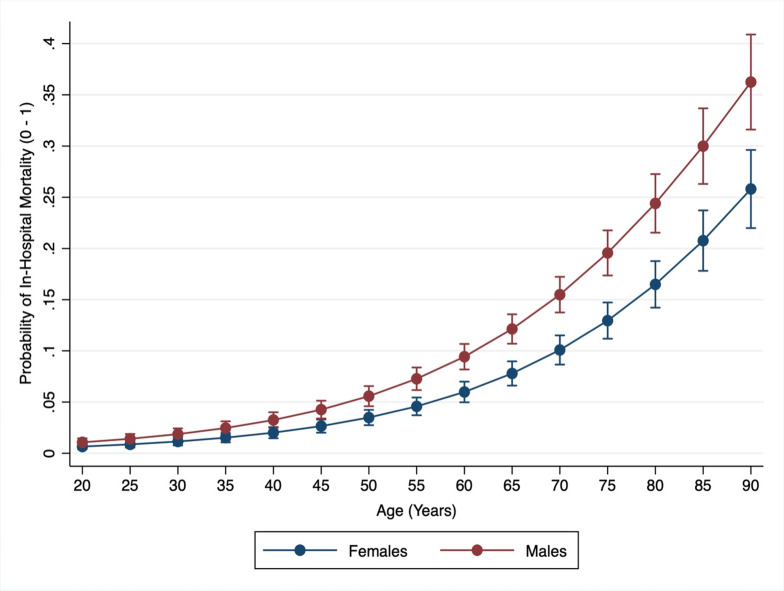
Predicted probabilities of COVID-19 mortality (Y-axis) by age (X-axis) among males and females. Predicted probably of SARS-CoV-2 infection for males (red line) and females (blue line) for age spectrum (20–90 years). Probability demonstrated on a scale of 0–1.

We did not observe a statistically significant interaction between age and sex for the outcomes of in-hospital mortality (p = 0.85). The AUC of our fully adjusted in-hospital mortality model was excellent (0.96) and model fit was optimal (p value for goodness of fit = 0.74). The likelihood of in-hospital mortality among males and females across the age spectrum among hospitalized COVID-19 patients is demonstrated in Fig 3. Excluding COVID-19 patients who were admitted at the time of our analyses, the overall LOS for hospitalized male COVID-19 patients, median (IQR): 5 (2–9) was longer as compared to females, median (IQR) LOS: 4 (2–8), median difference (CI) 1.0 (0.63–1.37). After adjusting for age, ethnicity, insurance, income, ICU admission and mechanical ventilation the estimated LOS for males continued to be significantly higher as compared to females, with a median difference (CI): 0.44 (0.11–0.78).

## Discussion

We report data from a large diverse US metropolitan area which has been an epicenter of SARS-CoV-2 infection during the surge of the COVID-19 pandemic. The Houston metropolitan area is considered to be one of the most diverse in the US. According to US census data (2019), compared to the overall US race / ethnicity distribution, the Houston metropolitan as a higher proportion of Hispanic / Latino population (18.5% US vs. 45.0% Houston), higher proportion of Black / African American population (13.4% vs. 22.6%) and lower proportion of non-Hispanic White population (60.1% vs. 24.4%). Given the demographic mix, particularly the rapidly growing Hispanic / Latino population, on the average, Houston area population is relatively younger as compared to the US average. As per US census data, 10.5% of Houston area population is ≥ 65 years, whereas 16.5% of US population falls into this age category. However, Houston precisely reflects the US sex distribution; proportion of females in Houston area is 50.1% compared to 50.8% for US (US Census Bureau population estimates for July 1, 2019).

Though initial reports from China and Europe provided preliminary evidence of poor COVID-19 outcomes among males, to our knowledge data from large and diverse US metropolitan areas is lacking. Such data are necessary to synthesize contextually relevant evidence regarding an independent association between male sex and poor COVID-19 outcomes. We analyzed a large population sample of > 95,000 tested and > 4,500 hospitalized individuals from one of the most diverse US cities. After adjusting for several key socio-demographic, clinical, laboratory, hospital course and treatment variables, we demonstrate a clear and strong independent association between male sex and higher SARS-CoV-2 susceptibility, greater likelihood of ICU admission, utilization of mechanical ventilation, and longer LOS–all clinical indicators of higher severity of the COVID-19 disease. These findings are congruent with the approximately 45% higher risk of in-hospital mortality among males as compared to females.

Our findings are in line with studies of COVID-19 in China that found that the disease disproportionally affects men and women [13]. Initial data indicate that men suffer from more severe disease and have higher mortality than women [4, 7, 14, 15]. Sex disaggregated data from 71 different countries published by Global 50/50 (accessed December 24, 2020), an initiative to promote gender equality in healthcare, indicate that overall case fatality ratio was significantly higher in men than in women [13]. Despite having the highest number of COVID-19 cases in the world, the United States has only partly sex disaggregated data as the Center for Disease Control has not reported these data from all states and counties uniformly. Biological sex and gender may also be linked to differences in the clinical manifestation of COVID-19, although studies on this topic have been limited [15]. Consistent with our study, emerging data has revealed sex-related differences in COVID-19 severity and morbidity, with male COVID-19 patients having an increased risk of admission, aOR (CI): 1.68 (1.45–1.90), and in-hospital mortality, aOR (CI): 1.87 (1.33–2.63) [16]. Understanding sex differences in disease severity, clinical characteristics and mortality is a fundamental step for improved disease management, prediction of poor outcomes and intervention strategies for both men and women.

The underlying mechanisms or drivers of the observed sex differences are yet to be determined. The initial data on sex disparities in severity and mortality could be driven by a higher presence of comorbidities (i.e. cardiovascular disease, hypertension, diabetes and chronic lung disease) and high-risk behaviors including smoking and alcohol use in men [13]. However, in our cohort, the disparities remained even after controlling for these potential confounders. Men and women are also known to respond differently to foreign and self-antigens, and sex differences in the immune response are well-documented [17]. Men are more susceptible to pathogens while females mount a stronger antigenic response to infection, vaccines, and self-antigens at the cost of a higher prevalence of autoimmune disorders [18]. Sex differences may exist in angiotensin converting enzyme (ACE) 2 receptor and the cellular serine protease TMPRSS2, which are responsible for viral entry and priming respectively. Although TMPRSS2 is predominantly expressed in prostate epithelium, expression also occurs in the airway epithelium [19]. A TMPRSS2 inhibitor was recently shown to block entry of the virus in vitro, thus demonstrating potential for utilization as an antiviral therapeutic [20]. Hospitalized patients with moderate SARS-CoV-2 infection have also been observed to have elevated levels of specific inflammatory cytokines and chemokines, with sex differences existing in these immune responses [8]. Female patients at baseline had a more robust T cell activation than males at baseline across age. Loss of T cell activation was correlated with older age in males, and the association between poor T cell response with worse disease outcomes was observed in males only. In further investigating immune responses to COVID-19, a recent study implicated changes in Kynurenic acid as a potential driver of sex-specific effects [21].

Limitations of our study include analysis of data from a single healthcare system across the greater Houston metropolitan area; therefore, our data may not be representative of a generalized population. Case ascertainment (SARS-CoV-2 positivity) is subject to sensitivity and specificity of the employed PCR testing platforms, a small proportion of individuals who were tested multiple times could have been misclassified based on approach of using first encounter information. Furthermore, our investigation focused on evaluating the comorbidity and clinical parameters that may underlie observed sex differences. While strong evidence in support of the role of biological pathways–particularly in the immune response–has been presented, future studies may additionally explore how socio-behavioral factors–such as alcohol consumption or participation in other high-risk behaviors–might influence health outcomes.

In light of these limitations, our work demonstrates a higher risk for SARS-CoV-2 prognosis and severe COVID-19 outcomes among males and underscores the importance of further investigating the biological pathways that may influence disease etiology. Sex is increasingly recognized as a modifier of disease [22], and its role with respect to SARS-CoV-2 and COVID-19 appears to be no exception. Although distinctions in the immune response may constitute one explanation for sex-specific differences, further study is warranted to identify more robust strategies for patient risk stratification and targeted treatment intervention.

## Supporting information

S1 Checklist STROBE statement—checklist of items that should be included in reports of cross-sectional studies. (PDF)

S1 Table Univariable analysis of factors associated with death among hospitalized COVID-19 patients. (DOCX)
