# Supplementary material for: Sex differences in susceptibility, severity, and outcomes of coronavirus disease 2019: Cross-sectional analysis from a diverse US metropolitan area
Source: PLoS One. 2021 Jan 13;16(1):e0245556. doi: 10.1371/journal.pone.0245556 (PMC7806140; doi:10.1371/journal.pone.0245556)
Supplement: S1 Table — (DOCX) [file pone.0245556.s002.docx]

**S1 Table.** Univariable analysis of factors associated with death among hospitalized COVID-19 patients

|  | **Total**  **(4,509)** | **Discharged Alive**  **(4,057)** | **Died**  **(452)** | **p-value** |
| --- | --- | --- | --- | --- |
| **Demographic and Social Factors** | | | | |
| Sex (Male) | 2,298 (51.0) | 2,031 (50.1) | 267 (59.1) | <0.001 |
| Age (Years) | 59.0 (17.5) | 57.6 (17.2) | 71.8 (13.8) | <0.001 |
| Race |  |  |  | 0.53 |
| White | 2,761 (61.2) | 2,483 (61.2) | 278 (61.5) |  |
| Black | 1,182 (26.2) | 1,067 (26.3) | 115 (25.4) |  |
| Asian | 212 (4.7) | 185 (4.6) | 27 (6.0) |  |
| Other | 354 (7.9) | 322 (7.9) | 32 (7.1) |  |
| Ethnicity | 1,701 (38.3) | 1,565 (39.1) | 136 (30.6) | <0.001 |
| Marital Status |  |  |  | <0.001 |
| Single | 1,287 (28.5) | 1,202 (29.6) | 85 (18.8) |  |
| Married / Partner | 2,372 (52.6) | 2,133 (52.6) | 239 (52.9) |  |
| Widowed / Separated | 708 (15.7) | 592 (14.6) | 116 (25.7) |  |
| Unknown | 142 (3.1) | 130 (3.2) | 12 (2.7) |  |
| Insurance Type |  |  |  | <0.001 |
| Commercial | 1,521 (33.7) | 1,461 (36.0) | 60 (13.3) |  |
| Medicaid | 267 (5.9) | 252 (6.2) | 15 (3.3) |  |
| Medicare | 1,698 (37.7) | 1,511 (37.2) | 187 (41.4) |  |
| Other | 255 (5.7) | 80 (2.0) | 175 (38.7) |  |
| Self-Pay | 768 (17.0) | 753 (18.6) | 15 (3.3) |  |
| Median Zip-based Income (USD) | 62,083  (47303-78487) | 61,446  (47303-78483) | 65,805  (47817-79869) | 0.068 |
| Median Income (High vs. Low) | 2,455 (55.0) | 2,237 (55.7) | 218 (48.7) | 0.005 |
| Population Density (Zip) | 3141.9  (1504 - 4384.8) | 3141.9  (1504 - 4384.8) | 2991.6  (1439.1 - 4110.7) | 0.021 |
| **Comorbidities / Behavioral Factors / Pre-existing Conditions** | | | | |
| Population Density (High vs. Low) | 1,977 (44.2) | 1,798 (44.7) | 179 (39.9) | 0.051 |
| Body Mass Index | 31.3 (8.2) | 31.5 (8.3) | 29.9 (7.7) | <0.001 |
| Smoking (Current / Past) | 1,112 (26.4) | 949 (25.0) | 163 (39.8) | <0.001 |
| Hypertension | 3,067 (68.0) | 2,661 (65.6) | 406 (89.8) | <0.001 |
| Hyperlipidemia | 2,054 (45.6) | 1,774 (43.7) | 280 (61.9) | <0.001 |
| Myocardial Infarction | 808 (17.9) | 606 (14.9) | 202 (44.7) | <0.001 |
| Congestive Heart Failure | 938 (20.8) | 735 (18.1) | 203 (44.9) | <0.001 |
| Peripheral Vascular Disease | 655 (14.5) | 518 (12.8) | 137 (30.3) | <0.001 |
| Cerebrovascular Disease | 693 (15.4) | 562 (13.9) | 131 (29.0) | <0.001 |
| Dementia | 449 (10.0) | 352 (8.7) | 97 (21.5) | <0.001 |
| Chronic Pulmonary Disease | 1,019 (22.6) | 883 (21.8) | 136 (30.1) | <0.001 |
| Rheumatic Disease | 167 (3.7) | 144 (3.5) | 23 (5.1) | 0.10 |
| Peptic Ulcer Disease | 136 (3.0) | 105 (2.6) | 31 (6.9) | <0.001 |
| Mild Liver Disease | 411 (9.1) | 356 (8.8) | 55 (12.2) | 0.017 |
| Moderate to Severe Liver Disease | 85 (1.9) | 63 (1.6) | 22 (4.9) | <0.001 |
| Diabetes Without Complications | 1,913 (42.4) | 1,659 (40.9) | 254 (56.2) | <0.001 |
| Diabetes With Complications | 815 (18.1) | 652 (16.1) | 163 (36.1) | <0.001 |
| Paraplegia / Hemiplegia | 115 (2.6) | 97 (2.4) | 18 (4.0) | 0.042 |
| Renal Disease | 442 (9.8) | 356 (8.8) | 86 (19.0) | <0.001 |
| Cancer | 393 (8.7) | 325 (8.0) | 68 (15.0) | <0.001 |
| Metastatic Carcinoma | 294 (6.5) | 247 (6.1) | 47 (10.4) | <0.001 |
| AIDS / HIV | 37 (0.8) | 35 (0.9) | 2 (0.4) | 0.35 |
| Stroke (Ischemic / Hemorrhagic) | 478 (10.6) | 393 (9.7) | 85 (18.8) | <0.001 |
| Mild Cognitive Impairment / Dementia | 463 (10.3) | 364 (9.0) | 99 (21.9) | <0.001 |
| Other Neurological Disorders | 389 (8.6) | 338 (8.3) | 51 (11.3) | 0.034 |
| Charlson Comorbidity Index | 3 (1-7) | 3 (1-6) | 7 (4.5-10) | <0.001 |
| **Vital Signs / Physiological Monitoring** | | | | |
| Systolic Blood Pressure | 132.8 (19.8) | 132.9 (19.6) | 132.0 (21.4) | 0.37 |
| Diastolic Blood Pressure | 72.4 (9.9) | 72.8 (9.8) | 69.3 (10.5) | <0.001 |
| Respiratory Rate ≥ 24 breaths / min | 909 (21.0) | 729 (18.8) | 180 (40.4) | <0.001 |
| Temperature ≥ 38 C | 206 (4.8) | 194 (5.0) | 12 (2.7) | 0.031 |
| Oxygen Saturation < 94% | 772 (17.9) | 589 (15.2) | 183 (41.0) | <0.001 |
| Positive CAM ICU Score | 641 (49.2) | 351 (37.9) | 290 (77.1) | <0.001 |
| **Laboratory Parameters** | | | | |
| White Blood Cell Count < 4000/microliter | 254 (5.6) | 249 (6.2) | 5 (1.1) | <0.001 |
| Lymphocytes < 20% | 3,019 (67.1) | 2,595 (64.2) | 424 (93.8) | <0.001 |
| Platelet Count 150,000 / microliter | 516 (11.5) | 398 (9.8) | 118 (26.1) | <0.001 |
| B-natriuretic peptide > 100 pg/ml | 1,068 (33.4) | 807 (29.1) | 261 (62.1) | <0.001 |
| Procalcitonin > 0.25 ng/ml | 776 (47.3) | 570 (41.5) | 206 (77.4) | <0.001 |
| Troponin ≥ 0.06 ng/ml | 834 (32.4) | 546 (25.4) | 288 (67.9) | <0.001 |
| Aspartate aminotransferase > 40 U/l | 2,164 (49.2) | 1,830 (46.3) | 334 (74.4) | <0.001 |
| Alanine aminotransferase > 40 U/l | 1,850 (42.1) | 1,631 (41.4) | 219 (48.9) | 0.002 |
| Total Bilirubin ≥ 1.2 mg / dl | 159 (3.9) | 110 (3.0) | 49 (11.2) | <0.001 |
| C-Reactive Protein >8.2 ng/ml | 3,606 (94.1) | 3,190 (93.4) | 416 (99.8) | <0.001 |
| Ferritin level > 3000 ng/ml | 221 (5.7) | 159 (4.6) | 62 (15.0) | <0.001 |
| D-dimer > 0.5 ug/ml | 3,211 (84.3) | 2,813 (82.6) | 398 (99.3) | <0.001 |
| Creatinine > 1.5 mg/dl | 768 (17.3) | 556 (13.9) | 212 (46.9) | <0.001 |
| Venous lactate > 2.2 mmol/l | 661 (20.7) | 447 (16.0) | 214 (51.8) | <0.001 |
| **Hospital Course / Complications** | | | | |
| Pneumonia | 3,805 (84.4) | 3,381 (83.3) | 424 (93.8) | <0.001 |
| Acute Respiratory Distress Syndrome | 427 (9.5) | 227 (5.6) | 200 (44.2) | <0.001 |
| Bronchitis | 121 (2.7) | 101 (2.5) | 20 (4.4) | 0.016 |
| Lower Respiratory Tract Infection | 99 (2.2) | 94 (2.3) | 5 (1.1) | 0.096 |
| Acute Renal Injury | 1,578 (35.0) | 1,221 (30.1) | 357 (79.0) | <0.001 |
| Acute Hepatic Injury | 123 (2.7) | 54 (1.3) | 69 (15.3) | <0.001 |
| Congestive Heart Failure | 857 (19.0) | 668 (16.5) | 189 (41.8) | <0.001 |
| Hypoxic Respiratory Failure | 2,525 (56.0) | 2,225 (54.8) | 300 (66.4) | <0.001 |
| **Medications** | | | | |
| Hydroxychloroquine | 372 (8.3) | 308 (7.6) | 64 (14.2) | <0.001 |
| Ribavirin | 103 (2.3) | 68 (1.7) | 35 (7.7) | <0.001 |
| Azithromycin | 673 (14.9) | 622 (15.3) | 51 (11.3) | 0.022 |
| Lopinavir / Ritonavir | 25 (0.6) | 16 (0.4) | 9 (2.0) | <0.001 |
| Remdesivir | 949 (21.0) | 850 (21.0) | 99 (21.9) | 0.64 |
| Tocilizumab | 740 (16.4) | 571 (14.1) | 169 (37.4) | <0.001 |
| Antithrombotic | 1,546 (34.3) | 1,305 (32.2) | 241 (53.3) | <0.001 |
| Anticoagulants | 4,070 (90.3) | 3,649 (89.9) | 421 (93.1) | 0.030 |
| Dexamethasone | 2,162 (47.9) | 1,914 (47.2) | 248 (54.9) | 0.002 |
| Donepezil | 135 (3.0) | 106 (2.6) | 29 (6.4) | <0.001 |
| Rivastigmine | 28 (0.6) | 22 (0.5) | 6 (1.3) | 0.044 |
| Galantamine | 8 (0.2) | 7 (0.2) | 1 (0.2) | 0.82 |
| Memantine | 102 (2.3) | 81 (2.0) | 21 (4.6) | <0.001 |
| **Acuity of Care Factors** | | | | |
| ICU Admit | 1,302 (28.9) | 926 (22.8) | 376 (83.2) | <0.001 |
| Mechanical Ventilation | 691 (15.3) | 373 (9.2) | 318 (70.4) | <0.001 |
